# Evaluation of waste paper for cultivation of oyster mushroom (*Pleurotus ostreatus*) with some added supplementary materials

**DOI:** 10.1186/s13568-020-0945-8

**Published:** 2020-01-18

**Authors:** Teklemichael Tesfay, Tesfay Godifey, Roman Mesfin, Girmay Kalayu

**Affiliations:** 1 Department of Biology, Aksum University, Tigray, Ethiopia; 2 Present Address: Department of Biology, Raya University, Tigray, Ethiopia

**Keywords:** Biological efficiency, Mycelia running, Maturation, Primordial initiation, Spawn, Yield

## Abstract

Mushroom cultivation is an economically feasible bio-technological process for conversion of various lignocellulosic wastes. The aim of this study was to evaluate the suitability of waste paper supplemented with corn stalk and wheat bran for oyster mushroom cultivation. Pure culture of Oyster mushroom (*Pleurotus ostreatus* (Jacq.: Fr.) Kummer) was purchased from YB Plant Micropropagation Plc; Mekelle, Ethiopia. Then, the pure culture was used as inoculum for spawn preparation using sorghum prepared in Microbiology laboratory, Department of Biology, Aksum University. Waste paper supplemented with corn stalk and wheat bran with 0%, 25% and 50% were prepared. The substrates were mixed with the spawn that has been inoculated with pure culture of oyster mushroom aseptically for their productivity and biological efficiency (BE) for cultivation of *P. ostreatus* mushroom. Data were analyzed using SPSS version 20. Higher (26.20 ± 19.36) mean weight, pileus diameter (7.90 ± 2.66 cm), total yield (646.4 ± 273.1 g) and BE (64.64 ± 273%) were obtained from waste paper (50%) supplemented with cornstalk (25%) and wheat bran (25%). And lower (17.92 ± 81.95%) BE were obtained from waste paper (100%). Moreover, the highest (3.88 ± 0.32 cm) mean stalk length was obtained from waste paper (50%) supplemented with corn stalk (50%). This study revealed that waste paper supplemented with corn stalk and wheat bran resulted in high BE and total yield. Thus, utilization of waste paper appears to be a promising alternative for the cultivation of oyster mushroom when supplemented with other substrates.

## Introduction

Mushrooms are fleshy, spore-bearing, multicellular fungi. Mushrooms are a good source of protein, vitamins and minerals and are known to have a broad range of uses both as food and medicine. Oyster mushroom, *Pleurotus ostreatus*, has been widely cultivated and commercialized next to *Agaricus bisporus*. Several studies have reported that *P. ostreatus* contains approximately 100 bioactive compounds, which is a potential source of dietary fiber. Besides, they are rich in protein, lipids, carbohydrates, vitamin and minerals content but low in calories and fat content (Deepalakshmi and Mirunalini 2014). Oyster mushroom are the easiest and least expensive commercial mushrooms to grow because they are well known for conversion of crop residues to food protein and are considered as potential source of income, alternative food production, provision of employment, and for recycling of agricultural wastes (Banik and Nandi 2004).

Oyster mushroom has abilities to grow at a wide range of temperatures utilizing various lignocelluloses (Sánchez 2010). Oyster mushrooms produce extensive enzymes and utilize complex organic compounds which occur as agricultural wastes and industrial by-products (Baysal et al. 2003). Thus, most organic matters containing cellulose, hemicellulose and lignin can be used as mushroom substrate i.e. rice and wheat straw, cottonseed hulls, corncob, paddy straw sugarcane baggase, sawdust, waste paper, and leaves (Sharma et al. 2013). However, an ideal substrate should contain nitrogen (supplement) and carbohydrates for rapid mushroom growth (Khare et al. 2010).

Mushroom cultivation in Ethiopia is a young project and is not expanded and familiarized in different parts of the country. Oyster mushroom cultivation can play an important role in alleviating food security and diversifying business and employment opportunities in the both urban and rural areas as most of Ethiopian (85%) depend on agriculture. Various substrates such as wheat straw, grass, rice straw, barley straw, and bean straw have been used for mushroom cultivation in different parts of the world. In Ethiopia, these substrates are used for animal feed in by farmers and not accessible by mushroom grower with reasonable price. The present study is an attempt to look for other locally available substrates. In Axum there are a number of offices and colleges dispose waste papers. Waste papers and corn stalk are disposed off in an open-field burning, which leads to environment pollution. Waste papers are composed of primarily cellulose, hemicellulose and lignin. If these can support the growth of oyster mushroom, it may help to transform these wastes into an accepted edible biomass of high market value, and serve as a cheap source of substrate for mushroom growers. Therefore, the current study was aimed at evaluating waste paper supplemented with cornstalk, and wheat bran as substrates for the cultivation of mushroom.

## Materials and methods

### Description of the study area

The study was conducted in Microbiology laboratory, Department of Biology, Aksum University, Axum, Ethiopia. Axum is located at altitude of 2133 m in the high lands of Tigray national and regional state in the north tip of Ethiopia at 14° 11′ N, and 38°73′ E. Axum’s average annual temperature is 18.3 °C and its average annual precipitation is 652 mm. The average annual relative humidity is 57.7% (National Metrology Agency 1992).

### Spawn preparation

For spawn preparation, 15 kg of sorghum was soaked in water overnight. The excess water was drained off and (5%) wheat bran and (2%) gypsum were added. The ingredients were thoroughly mixed and moisture was adjusted to 55–60%. Then the mixture was distributed equally into 250 ml sterile plastic bags, at the rate of 250 g sorghum seed per plastic bag and autoclaved, at 121 °C for 30 min. After cooling, each sterile plastic bag was inoculated with oyster mushroom culture (*Pleurotus ostreatus* (Jacq.: Fr.) Kummer; YB Plant Micropropagation Plc; Mekelle, Ethiopia). When the mixture was totally invaded by mycelium, after 15 days of incubation at 25 °C, the spawn was ready to be used for the inoculation of the solid substrate (Fan et al. 2000).

### Oyster mushroom cultivation techniques

Oyster mushroom cultivation was done according to (Randive 2012). Table 1 shows the compositions of substrates used as a treatment groups for the cultivation of oyster mushroom.

**Table 1:** Substrate composition of the treatment groups used for the cultivation of oyster mushroom

Treatment group	Composition of the substrate	Number of replicas of each trial performed
T1	Waste paper (50%) + corn stalk (50%)	Five
T2	Waste paper (75%) + corn stalk (25%)	Five
T3	Waste paper (50%) + corn stalk (25%) + wheat bran (25%)	Five
T4	Waste paper (100%)	Five
T5	Waste paper (75%) + wheat bran (25%)	Five
T6	Cotton husk (control)	Five

*T* treatment

Initially, waste paper and corn stalk were chopped using edge tool into small pieces (3–5 cm long). The substrates were soaked in water for 24 h to moisten it thoroughly and pasteurized using clean steel drums. First the water was heated at 60 °C. Then the substrate was added and allowed to remain in the water for 30 min. Finally, once pasteurized, it was stalked in sterile plastic sheet (2 m × 4 m) laying on the steep cemented floor so as to remove the excessive moisture from the substrates to get 65–75% moisture level. Then, the spawn prepared was mixed with substrate in the sterile plastic sheet (2 m × 4 m) laying on the floor. The mixture was filled into 5000 ml sterile plastic bags and placed in dark room. Holes were prepared for aeration in the plastic bag. Once the mycelia colonized substrates in plastic bags, the bags were transferred to the cropping room. The cropping room had a limited light with average temperature of 25 °C. The growing mycelia were watered twice a day (morning and afternoon) using watering pot to maintain water activity of the substrates in the plastic bags and humidity of cropping room.

### Harvesting and yield measures

Mature mushrooms were picked by clean hand without harming the substrate when they started to wrinkle-ripe. This was done for three subsequent flushes. Following the method of Iqbal et al. (2005), the yield parameters were recorded with respect to time (days) taken for completion of spawn running, time taken for the first appearance of pinhead formation, time taken for maturity of fruit bodies, number of flushes, and yield of flushes on the treatment substrates (total weight of all the fruiting bodies harvested from all the three pickings were measured and considered as total yield of mushroom). The pileus diameter and the stipe length were measured with graduated transparent ruler. Mature mushrooms were weighed with analytical balance to determine the biological efficiency (BE) of mushrooms produced from substrates. The average Biological efficiency (BE) of harvests was computed (Peng et al. 2000).

$$\text{BE = }\frac{{\text{Weight}\,\text{of}\,\text{fresh}\,\,\text{mushroom}\,\,\text{harvested}\,\,\text{per}\,\text{bag}\,\,{ \times }\,\,{100\% }}}{{\text{Weight}\,\text{of}\,\text{dry}\,\text{substrate}\,\,\text{per}\,\text{bag}\,\,\text{before}\,\,\text{inoculation}}}.$$

Mycelium running—time required from inoculation to completion of mycelium running were recorded (days). Primordial initiation—time required for primordial initiation (days) were recorded. Maturity—time required from primordial initiation to harvest (days) were recorded. Number of flushes—the numbers of flushes were counted in each plastic bag. Average weight of individual fruiting body/plastic bag**—**average weight of individual fruiting body was calculated as:

$$\text{Weight}\,\text{ = }\,\frac{{\text{Total}\,\text{weight}\,\text{of}\,\text{fruiting}\,\text{body}\,\text{per}\,\text{plastic}\,\text{bag}}}{{\text{Total}\,\text{n}\text{umber}\,\text{of}\,\text{fruiting}\,\text{body}}}.$$

Pileus diameter was measured by using a string passing from one end of the pileus to the other through the center of the pileus. It was obtained on three randomly picked mushrooms and then the average was calculated in millimeters (mm). Stipe length was measured by placing the string from one end, where it was attached to the substrate, to the point where the gills on the pileus start. The string was placed along a ruler to get the length in millimeters (mm).

Yield of mushroom= total weight of all the fruiting bodies harvested from all the three pickings were measured as total yield of mushroom.

The data on spawn running was recorded after complete colonization of substrate and pin head and fruit body formation were observed.

### Data collection and data analysis

Data on the mycelium colonization period, pin head formation period, stalk length, BE, step length, pileus diameter were recorded and analyzed using SPSS version 20. Analysis of variance (ANOVA) was used to indicate significant mean differences at 95% confidence interval using tukes multiple comparisons method.

## Results

The number of days taken for complete mycelial growth differs significantly (p < 0.05) among the treatments. In the current study, the fastest mycelia extension was observed in T1 (15 days), T3 (15 days), and T5 (15 days) (Table 2). T2 and T4 took the maximum numbers of days (21 and 17), respectively.

**Table 2:** The effect of substrates on mycelium colonization period (days)

Substrate	Mean ± SD			Overall mean ± SD
	Flush 1	Flush 2	Flush 3	
T1	15^a^	15^a^	15^a^	15^a^
T2	21 ± 1.6^b^	21 ± 1.6^b^	21 ± 1.6^b^	21 ± 1.6^b^
T3	15^a^	15^a^	15^a^	15^a^
T4	17^a^	17^a^	17^a^	17^ac^
T5	15^a^	15^a^	15^a^	15^a^
T6	15^a^	15^a^	15^a^	15^a^

Mean values followed by different superscript letters within a row and columns are significantly different (p < 0.05)

Table 3 shows that the mean pin head formation of some of the treatments varied significantly (p < 0.05). Moreover, there is variation in pin head formation between flushes of each treatment. Time taken for initial appearance of pinhead after spawning of the substrate was 9.46 ± 0.8 and 11.60 ± 3.24 days for treatment three and five, respectively. Thus, treatment three and five showed a better performance in case of pin-head formation.

**Table 3:** The effect of substrates on pin head formation

Substrate	Mean ± SD and 95% CI of pin for each flush			Overall mean ± SD
	Flush 1	Flush 2	Flush 3	
T1	13 ± 1.22^a^	18.2 ± 1.30^b^	16.2 ± 1.09^bc^	15.8 ± 2.48^a^
T2	12.8 ± 0.84^a^	15.60 ± 2.30^a^	17.20 ± 2.16^a^	15.20 ± 2.56^a^
T3	5.80 ± 1.30^a^	10.80 ± 0.86^b^	11.80 ± 1.64^bc^	9.46 ± 0.80^b^
T4	19 ± 2.34^a^	16.20 ± 5.67^a^	14.40 ± 3.20^a^	16.53 ± 4.18^a^
T5	14.60 ± 2.79^a^	9.80 ± 3.03^b^	10.40 ± 1.67^ac^	11.60 ± 3.24^C^
T6	8	10	9	9.0 ± 1.00^d^

Mean values followed by different superscript letters within a row and columns are significantly different (p < 0.05)

Table 4 indicates mean ± SD for each flush and the overall maturity (days) of oyster mushroom. Maturity was not significantly different (p > 0.05) among the flush of each treatment, while among the treatments, T4 (Waste paper 100%) was significantly (p < 0.05) different. Considering the minimum number of days taken for maturity of fruiting bodies, T1 (3.4, 3.6 and 3.4 days) appears to be the best substrate followed by T3 (4.2, 3.6 and 3.2 days) (Table 4). Maximum time period (4.4, 4.4, 4 days) was required for the maturity of fruiting bodies in case of T4 (waste paper (100%)). Besides, maturity between treatments was not significantly different. The mean maturity of the different treatments ranged from 3.47 ± 0.52 (T1) to 4.27 ± 0.88 (T4). However, it took less days for maturation compared to the control group.

**Table 4:** Period of pinning-to-maturation of mushrooms in substrates (days)

Substrate	Mean ± SD and 95% CI of maturity for each flush			Overall mean ± SD
	Flush 1	Flush 2	Flush 3	
T1	3.40 ± 0.55^a^	3.60 ± 0.55^a^	3.40 ± 0.548^a^	3.47 ± 0.52^a^
T2	3.60 ± 0.55^a^	3.80 ± 0.45^a^	3.80 ± 0.45^a^	3.73 ± 0.46^a^
T3	4.20 ± 0.84^a^	3.60 ± 0.55^a^	3.20 ± 0.45^a^	3.67 ± 0.72^a^
T4	4.40 ± 0.89^a^	4.40 ± 0.89^a^	4.00 ± 1.00^a^	4.27 ± 0.88^b^
T5	4.00 ± 0.71^a^	3.60 ± 0.55^a^	3.60 ± 0.55^a^	3.73 ± 0.59^a^
T6	4^a^	4^a^	6^a^	4.53 ± 0.74^a^

Mean values followed by different superscript letters within a row and columns are significantly different (p < 0.05)

Table 5 indicates that higher mean stalk length was measured in T1 (3.88 ± 0.32 cm) followed by treatment three (3.62 ± 0.36 cm). However, no significant difference was observed in terms of stalk length between the different substrates and the control. But, a decreasing pattern was observed in terms of stalk length of flush in each treatment.

**Table 5:** The effect of substrate on stalk length (cm)

Substrate	Mean ± SD and 95% CI of stalk length for each flush			Overall mean ± SD
	Flush 1	Flush 2	Flush 3	
T1	5.32 ± 0.17^a^	3.72 ± 0.29^b^	2.60 ± 0.14^c^	3.88 ± 0.32^d^
T2	4.40 ± 0.69^a^	3.72 ± 0.48^a^	2.44 ± 0.59^a^	3.52 ± 0.38^d^
T3	4.98 ± 0.46^a^	3.12 ± 0.41^b^	2.76 ± 0.51^bc^	3.62 ± 0.36^d^
T4	3.60 ± 0.28^a^	3.16 ± 0.21^a^	2.26 ± 0.19^ab^	3.01 ± 0.19^d^
T5	4.12 ± 0.38^a^	3.30 ± 0.44^a^	3.32 ± 0.55^a^	3.58 ± 0.26^d^
T6	4.62^a^	3.64^a^	3.00^a^	3.75 ± 0.47^d^

Mean values followed by different superscript letters within a row and columns are significantly different (p < 0.05)

Table 6 shows that the mean ± SD of each flush and the overall mean of pileus diameter. The highest (7.90 ± 2.66 cm) and the lowest (5.4

**Table 6:** The effect of substrates on Pileus diameter (cm)

Substrate	Mean ± SD and 95% CI of pileus diameter for each flush			Overall mean ± SD
	Flush 1	Flush 2	Flush 3	
T1	7.30 ± 0.42^a^	7.04 ± 0.74^a^	6.32 ± 1.04^a^	6.88 ± 0.84^a^
T2	9.08 ± 2.29	7.26 ± 1.82^a^	5.24 ± 1.41^b^	7.19 ± 2.37^a^
T3	10.58 ± 2.40^a^	7.16 ± 0.76^a^	6.16 ± 0.76^b^	7.90 ± 2.66^ac^
T4	6.08 ± 0.95^a^	6.34 ± 0.97^a^	5.12 ± 0.26^a^	5.85 ± 0.92^ab^
T5	6.28 ± 1.19^a^	5.50 ± 1.98^a^	4.42 ± 1.09^a^	5.40 ± 1.57^a^
T6	6.30^a^	6.30^a^	9.10^a^	7.23 ± 1.61^a^

Mean values followed by different superscript letters within a row and columns are significantly different (p < 0.05)

0 ± 1.57 cm) mean pileus diameter were noted on T3 and T5, respectively. Significant difference was observed between T3 and T4. Besides, pileus diameter among flushes was not significantly (p < 0.05) different except in the second and third flushes of T2 and T3 (Table 6).

Table 7 indicates the effect of substrate on mushroom weight (g). Of the 1st flush generation, the maximum (34.08 ± 45.69 g) and minimum (6.34 ± 1.44 g) mean weight (g) were recorded on T2 and T1 respectively. Of the 2nd flush generation, the highest mean weight (33.76 ± 22.47) was recorded on T4. Mean weight of harvested flush decrease with successive generations (Table 7). Besides, the higher (26.20 ± 19) value of overall mean weight of individual fruiting body was observed in T3.

**Table 7:** The effect of substrates on weight (g)

Substrate	Mean ± SD and 95% CI of weight for each flush			Overall mean ± SD
	Flush 1	Flush 2	Flush 3	
T1	6.34 ± 1.44^a^	4.94 ± 1.71^a^	5.08 ± 2.31^a^	5.45 ± 1.84^a^
T2	34.08 ± 45.69^a^	14.04 ± 12.38^a^	15.36 ± 4.97^a^	21 ± 27.15^a^
T3	33.90 ± 1.06^a^	32 ± 24.92^a^	12.70 ± 15.71^a^	26.20 ± 19.36^b^
T4	21.16 ± 11.15^a^	33.76 ± 22.47^a^	14.56 ± 11.00^a^	23.16 ± 16.80^a^
T5	28.20 ± 7.46^a^	24.64 ± 20.16^a^	21.38 ± 13.38^a^	24.74 ± 13.84^b^
T6	27.4^a^	19.7^a^	21.33^a^	22.80 ± 4.06^a^

ª The mean difference is significantly at 0.05 level

Table 8 indicates the effect of the treatment groups with varying substrate composition on yield (g) and BE (%). The highest total yield (682.1 g) was obtained from control followed by T3 (646.4 ± 273.1 g). Of the 1st flush harvested, the maximum yield (435.86 ± 133.34 g) was recorded on T3, while the lowest (87.4 ± 48.07) yield was obtained from T4. On the other hand, in 2nd generation flush, the mean yield ranged from 57.40 ± 15.85 (g) to 232 g and the highest was recorded on cotton husk (control). In the 3rd generation flush the minimum (34.40 ± 18.06 g) total yield was recorded on treatment four. Though, significant (p < 0.05) difference was observed only in T4 ignoring the flushes.

**Table 8:** The effect of substrate on yield (g) and BE (%)

Substrate	Mean ± SD and 95% CI of yield for each flush			Overall mean ± SD	Total yields (g)	Biological efficiency (BE) (%)
	Flush 1	Flush 2	Flush 3			
T1	324.72 ± 1.98^a^	225 ± 23.81^b^	70.28 ± 27.28^c^	206.68 ± 111.39	620.04 ± 83.07^a^	620.04 ± 83.07^a^
T2	331 ± 33.03^a^	95.72 ± 37.47^b^	57.06 ± 34.81^bc^	161.26 ± 129.46	483.78 ± 105.31^a^	48.44 ± 105.31^a^
T3	435.86 ± 133.3^a^	140.92 ± 65^b^	69.62 ± 74.84^bc^	215.46 ± 186.59	646.4 ± 273.1^a^	646.4 ± 273.1^a^
T4	87.4 ± 48.07^a^	57.40 ± 15.85^ab^	34.40 ± 18.06^bc^	59.73 ± 36.46	179.2 ± 81.95^d^	179.2 ± 81.95^d^
T5	243.8 ± 200.5^a^	163.8 ± 178.47^a^	115.2 ± 132.46^a^	174.26 ± 169.15	522.8 ± 511.4^a^	522.8 ± 511.4^a^
T6	302.5^a^	232^a^	147.6^a^	227.36 ± 77.55	682.1^a^	68.21^a^

Mean values followed by different superscript letters within a row and columns are significantly different (p < 0.05)

## Discussion

Mycelial growth provides suitable internal conditions for fruiting (Appendix Fig. 1a). In this study, the fastest mycelia extension was observed in T1 (15 days), T3, and T5 equally. Thus, outstanding growth of mycelium is a vital factor in mushroom cultivation (Pokhrel et al. 2009). In this study, waste paper supplemented with wheat bran and corn stalk produced mycelium extension within short period of time which is similar with the control except the T2. However, waste paper without supplementary materials took relatively extended time. This variance could be due to the variation in nutrient content, lignin and cellulose composition and moisture holding capacity of the substrate. Similar results were reported by Shah et al. (2004) who the growth of *Pleurotus* species on wheat straw, rice husk as well as saw dust took 2–3 weeks for mycelial growth after inoculation. Moreover, Kumari and Achal (2008) noted that colonization of the substrate with *P. ostreatus* was completed within 20 days of inoculation. Conversely, the current study contradicts with the results of (Girmay et al. 2016) who reported that mycelia running on waste paper took 14 days. The variation in mycelia extension might be due to the difference in condition of the environment and the nature of the substrate. *P. ostreatus* grew quickly at 30 °C (Marino et al. 2003) and oyster yield decreases when the temperature decreases in different climatic zones (Zervakis et al. 2001).

Shah et al. (2004) indicated that relatively higher room temperature resulted primordial initiation ranged 27 to 34 days of incubation. Moreover Sharma et al. (2013) reported shorter pining period (26.40–31.60 days of incubation) on various substrates. In the present study, all the treatments initiated the pin head within few days (ranged 9–16 ± 4) (Appendix Fig. 1b). Oei (2003) reported that materials with high quality lignin and cellulose contents take longer time to start pinning compared to the substrates with low contents of the lignin and cellulose. This study reveals that as the amount of waste paper increases the time taken for pinning increases (Table 3). Thus, the longer time taken for pinning might be due to the cellulose and lignin content of waste paper. Different scholars (Shah et al. 2004; Sharma et al. 2013) reported different pinning days. The variation in pin head formation might be due to the difference in room temperature of the cultivation room and nutrient availability of the substrate (Oei 2003; Shah et al. 2004; Sharma et al. 2013).

A number of investigators have reported different timing period for fruiting bodies (maturity). Similar results (4 ± 0.7 days) for the maturation of fruit bodies were reported by Gume et al. (2013). Appendix 2 exhibits cultivated fruiting body of oyster mushroom in the present study. The current result is also comparable with Islam et al. (2009) who reported that maturation period of *Pleurotus* species ranging from 3.29 to 4.33 growing on saw dusts of Mango, Shiris, Jackfruit, Kadom, Jam and Coconut. Moreover, higher (27–40) number of maturation days of *P.ostreatus* mushroom cultivated on waste paper has been reported (Girmay et al. 2016). This variation in maturity of fruiting bodies could be owing to the difference in physiological requirements and the nature of the substrate (Girmay et al. 2016).

Gume et al. (2013) reported shorter (1.4–1.9 cm) stalk length and pileus diameter (3.8–5.2 cm) than the current finding on mushrooms grown on sawdust, coffee bean husks, and corncobs. Stipe (stalk) length and pileus diameter of oyster mushroom grown in different substrates depend on the structure, compactness and physical properties of the substrate which in turn depends on the type of substrates. The substrates with higher moisture retaining capacity perform better than those with lower moisture retaining capacity (Chukwurah et al. 2013). Fruit bodies with larger pileus (caps) and shorter stipes (stalk) are better than that with smaller pileus and longer stipes (Synytsya et al. 2008). In the current study, treatment two provided better quality of mushroom with larger pileus diameter and shorter stalk length. However, the stipes contained more insoluble dietary fibers that can be used for the preparation of biologically active polysaccharide complexes utilizable as food supplements than pilei. Moreover, Kivaisi et al. (2003) have indicated that the size of the pileus depends on the aeration and amount of light.

Sarker et al. (2007) reported that the individual weight of fruiting body ranged from 1.33–1.59 g, which was less than the current finding. Similarly, Bhuyan (2008) has reported less (5.02–7.01 g) weight than the current study. Moreover, the author further remarked that a significant effect of supplementation on weight of individual fruiting bodies. The variation in weight of individual fruiting bodies might be due to environmental conditions or growing season and variation in nutrient composition of the substrates.

Yield among the flushes of each treatment varied significantly (p < 0.05) for some of the treatments (Table 8). Besides, the yield of all the treatments did not varied significantly (p > 0.05). This indicates that waste paper supplemented with corn stalk and wheat bran could replace cotton husk for cultivation of mushroom. In this research, higher yield was obtained compared to Sharma et al. (2013) with 381.85 g yield of *Pleurotus ostreatus* growing on rice straw, rice straw + wheat straw, rice straw + paper, sugarcane bagasse and sawdust of alder.

Biological efficiencies (BE), the conversion efficiency of substrate in mushroom cultivation, was computed as the ratio of the fresh mushroom harvested per bag to the dry weight of each substrate.The maximum biological efficiency (64.64 ± 273.1) was recorded on T3, while the lowest (17.92 ± 81.95%) BE was obtained from T4. This is in line with the works of Holkar and Chandra (2016) who reported that the biological efficiencies of *P.ostreatus* growing on wheat straw ranged from 63.4 to 74. As per Gume et al. (2013), substrates that gave over 40% BE could be recommended for oyster mushrooms cultivation. Thus, the current study reveals that all the treatments except 100% waste paper (T4) gave higher BE (Table 8). This could be due to the better availability of nitrogen, carbon and minerals from the supplements (Shah et al. 2004).

This study clearly indicates that waste paper supplemented with corn stalk and wheat bran offers higher total yield and biological efficiency. It represents promising substrates which can serves as a basal medium for the cultivation of oyster mushroom. It appears that the lignin, cellulose and hemicellulose, the active components of paper, provides a carbon sources. Thus, it is ecofriendly approach in terms of solid waste management and is also economically sound in light of food security.

## Figures and Tables

**Fig. 1:**
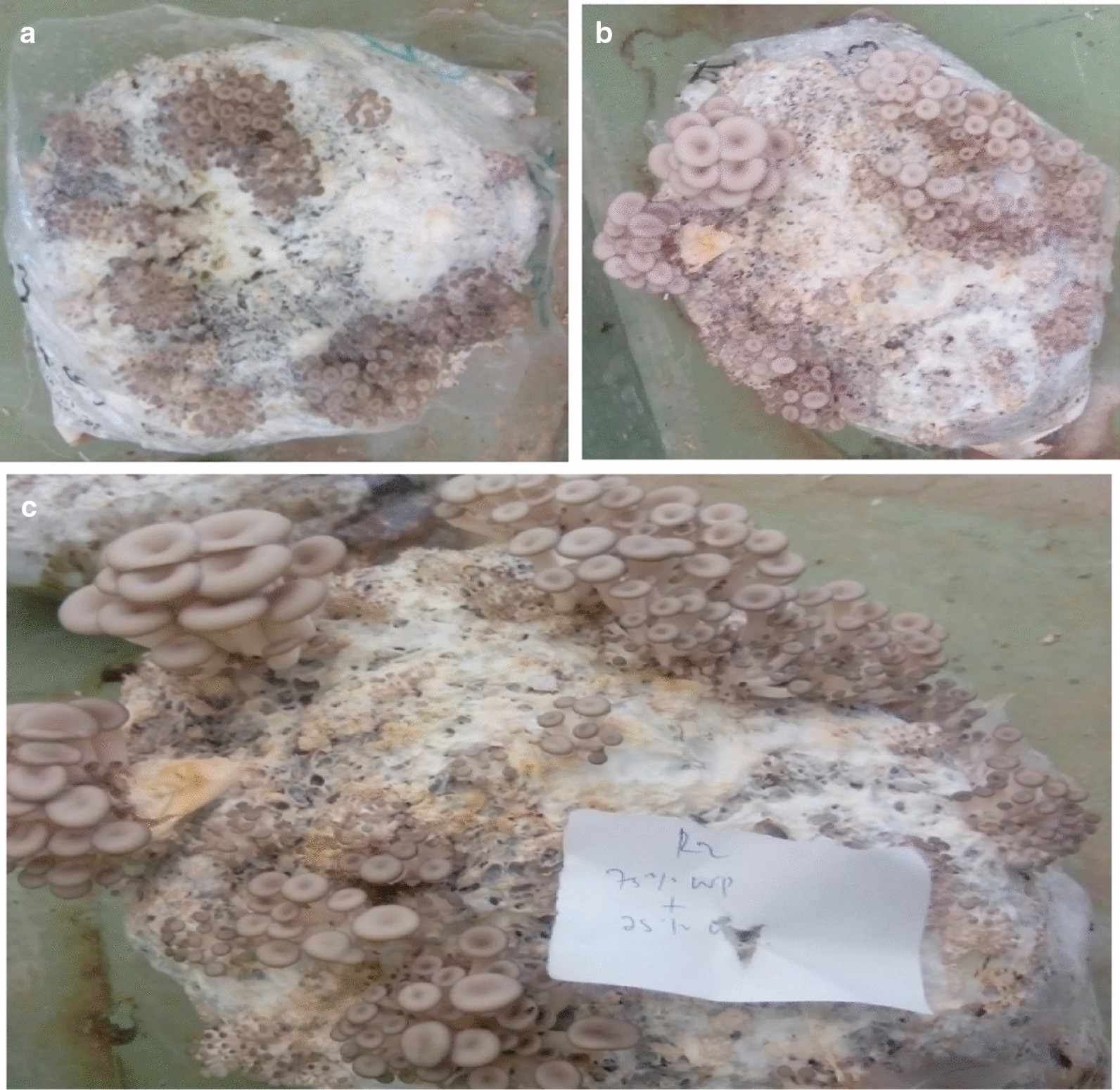
Mycelia running (**a**), pin head formation (**b**), pining to maturation (**c**)

**Fig. 2:**
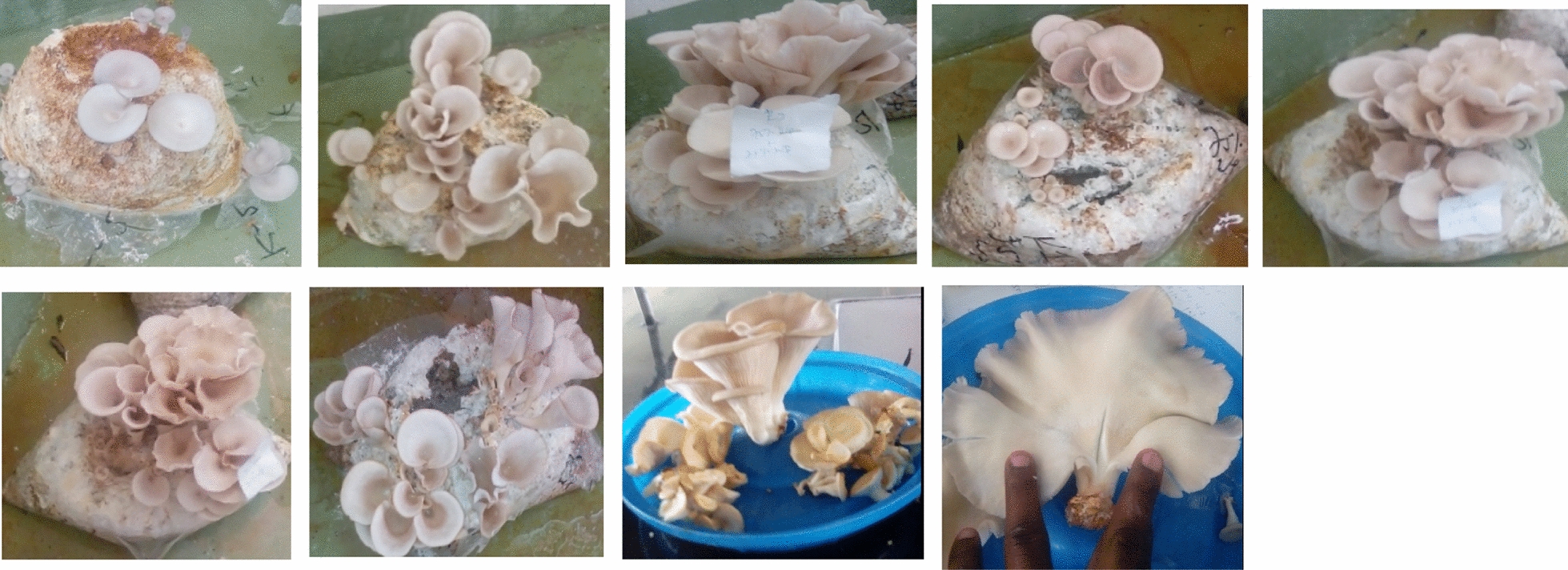
Cultivated fruiting body of oyster mushroom on different substrate composition

## Data Availability

The datasets supporting the conclusions of this article are included within the article.

## Appendices

### Appendix 1

See Fig. 1.

### Appendix 2

See Fig. 2.
